# Exploring the potential of hydro alcoholic crude extract of beeswax as antibacterial antifungal antiviral antiinflammatory and antioxidant agent

**DOI:** 10.1038/s41598-025-17830-4

**Published:** 2025-09-12

**Authors:** Alsayed E. Mekky, Nehad M. El-Barkey, Hesham M. Abd El Halim, Sara A. Nasser, Nashaat N. Mahmoud, Abdullah A. Zahra, Mohamed A. Nasr-Eldin

**Affiliations:** 1 Department of Botany and Microbiology, Faculty of Science, Al-Azhar University, Cairo, 11884 Egypt; 2 Entomology Department, Faculty of Science, Benha University, Benha, 13511 Egypt; 3 Department of Plant Protection, Faculty of Agriculture, Al-Azhar University, Cairo, Egypt; 4 Department of Botany and Microbiology, Faculty of Science, Benha University, Benha, 13511 Egypt

**Keywords:** *Apis mellifera* L., Anti-inflammatory, Antimicrobial, Antiviral, Antioxidant, Cytotoxicity, Phenolic compounds, Biological techniques, Biotechnology

## Abstract

*Apis mellifera* beeswax is synthesized from honey sugars and secreted by specialized glands located on the ventral side of the abdomen of worker bees aged 12–18 days. This beeswax possesses various therapeutic properties beneficial to human health. Given the priority in pharmacological research to develop new drugs from natural sources with minimal side effects, the absence of studies on the biological activities of *A. mellifera* beeswax is notable. Therefore, this study aims to investigate the chemical composition, as well as the anti-inflammatory, antiviral, cytotoxic, antimicrobial, and antioxidant activities of hydroethanolic crude beeswax extract from *A. mellifera*. The results revealed the presence of the flavonoids rutin (19.39 µg/g), naringenin (69.52 µg/g), daidzein (3.34 µg/g), quercetin (53.69 µg/g), kaempferol (51.88 µg/g), hesperetin (8.13 µg/g) and the phenolic gallic acid (421.13 µg/g), chlorogenic acid (878.80 µg/g), methyl gallate (56.21 µg/g), caffeic acid (8.83 µg/g), syringic acid (8.07 µg/g), coumaric acid (9.80 µg/g) and cinnamic acid (4.94 µg/g) which assessed by HPLC.* Apis mellifera* beeswax had considerable anti-inflammatory activity percent hemolysis Inhibition was 90.2% at concentration 1000 µg/mL, while indomethacin (standard drug) showed 100% activity at the same concentration. Also, exhibited major antioxidant activity as assessed by DPPH radical scavenging activity assay which compared to ascorbic acid, the IC_50_ of *A. mellifera* beeswax was 16.93 µg/mL. The antiviral activity of *A. mellifera* beeswax against HAV was recorded at 78.52%, while it was 24.2% against CoxB4 at the same concentration. The beeswax of *A. mellifera* demonstrated greater inhibitory activity than the reference compound gentamicin against *Staphylococcus aureus* (25 ± 0.18 mm) and *Escherichia coli* (15 ± 0.81 mm). It also showed significant inhibitory activity against *Candida auris* (22 ± 0.21 mm). Moderate inhibitory effects were observed against *Klebsiella pneumoniae* (19 ± 0.36 mm), *Enterobacter aerogenes* (14 ± 0.46 mm), and *Candida albicans* (15 ± 0.13 mm). The minimum lethal concentrations and minimum inhibitory concentrations of *A. mellifera* beeswax against these pathogenic strains ranged from 125 to 250 μg/mL and 62.5–125 μg/mL, respectively. The findings of this study highlight the substantial biological potential of beeswax extract, suggesting that *A. mellifera* beeswax warrants further investigation as a source for new pharmacological alternatives. However, additional in vitro and in vivo studies are necessary to fully assess its potential benefits for human health.

**Supplementary Information:**

The online version contains supplementary material available at 10.1038/s41598-025-17830-4.

## Introduction

Since ancient times, beekeeping products have been widely used in folk medicine because of their strong curative abilities and high amounts of bioactive molecules. These products include honey, propolis, pollen, royal jelly, beeswax, and bee venom^1^. Apitherapy, a now scientifically grounded branch of traditional medicine, employs apitherapy to prevent or treat various conditions such as wounds, rheumatoid arthritis, neurological and immunological conditions, and digestive tract problems, among just a handful^2,3^. Wax is one of the goods produced by bees that people particularly value and use frequently. The most widely cultivated species by humans, *Apis mellifera* and *Apis cerana*, provide the most widely used wax, beeswax, making this useful bee product more easily accessible.

Beeswax is secreted by worker bees through wax glands in their abdomens. Beeswax is produced mostly in late spring, during the colony expansion period, and is used to make combs^4^. The crystalline form of beeswax, which is produced from honey sugars, makes it perfect for beehive construction. Its chemical makeup varies depending on the species of bee and the area in which it is found. It is composed of hydrocarbons, free fatty acids, free fatty alcohols, linear wax monoesters, hydroxymonoesters made from palmitic, 15-hydroxypalmitic, and oleic acids, and complex wax esters that contain diols and 15-hydroxypalmitic acid^4,5^.

Currently, *A. mellifera* L., the honeybee, is the dominant species in the world beekeeping industry. This article, however, is on a different type of bee found in the family Hymenoptera and subfamily Meliponinae. Known as “stingless bees” because of their drastically decreased stings, which they utilize to defend their nests rather than sting people^6,7^. An estimated 40–90% of wild and cultivated plant species in the tropics are pollinated by stingless bees, making them an essential component of pollination processes^7^. Almost 600 species (61 genera) of the Meliponini tribe are found in tropical locations around the planet, mainly in the Neotropics, South and Central America, tropical Africa, Southwest Asia, and Australia^7,8^. The stingless bee produces and stores far less honey per hive (1–5 kg) than the dominant honey producers, *A. mellifera* bees, who generate an average of 20 kg of honey per hive. As a result, there are no quality control requirements, poor industrial production levels, and less awareness of their honey^6^. However, in contrast to *A. mellifera* honey, stingless bee honey has gained notice recently for its distinctive qualities, such as its unusual flavor and increased possibility of containing antimicrobial chemicals obtained from plants because of increased exposure to propolis^9^.

In 150 B.C., the famous Greek physician Galen created the first cosmetic lotion, which used beeswax as a major ingredient. This cream had an emulsion of water or rose water, olive oil, and beeswax^10^. Beeswax, often referred to as Madhuchishtha, is an important component in Ayurvedic medicine, an age-old and conventional Indian medical practice^11^. The increasing interest in natural products in Western countries, both as adjuncts to pharmaceuticals and as potential replacements, has led to a resurgence of interest in Ayurvedic medicine. Madhuchishtha (beeswax) is used topically for treating wounds from abrasions or burns and has been particularly effective in healing cracked heels^12^. Some reports also highlight its use in combination with other natural substances such as Madhu (honey), Guda (jaggery), or Taila (oil)^13^. Currently, beeswax is extensively studied and utilized in human medicine.

The objective of this investigation is to ascertain the phenolic profile and evaluate the hydroalcoholic crude extract of beeswax of *A. mellifera* L.’s in vitro antibacterial, anti-inflammatory, antiviral, cytotoxic, and antioxidant properties.

## Material and methods

### Chemicals

Sigma Chemical Co., Ltd. (St. Louis, MO, USA) provided all the chemicals including hydroalcoholic acid, methanol, gentamicin, amphotericin B, NaCL, MTT, 2,2-diphenyl-1-picrylhydrazyl, ascorbic acid, and indomethacin as well as other chemicals that needed for the analyses.

### Preparation of beeswax extract

In May 2023, beeswax from *A. mellifera* L. was acquired from Al-Azhar University’s Faculty of Agriculture in Nasr City, Egypt. The beeswax was ground into a powder and passed through a 50-mesh screen after being oven-dried at 45 °C. 200 g of beeswax were extracted in a Soxhlet apparatus with 1000 mL of 70% ethanol to get the hydroalcoholic crude extract (HCE). The filtrate was then concentrated using a rotary evaporator set at 40 °C. The concentrate was lyophilized and then stored in a tightly closed brown container at 5 °C until analysis.

### HPLC analysis

The beeswax sample’s (100 μg/mL) phenolic components were subjected to an HPLC analysis as follows: Agilent Technologies Inc., Santa Clara, CA, USA, provided the Agilent 1260 series HPLC system for the analysis of the beeswax extract. A C18 column (100 mm × 4.6 mm i.d., 5 µm) was used to pass the separation. The mobile phase was made up of (A) acetonitrile at 0.6 mL/min; (B) methanol; and (C) water with 0.2% H_3_PO_4_. The following method was used to determine the gradient elute: 0–11 min (96% A, 2% B); 11–13 min (50% A, 25% B); 13–17 min (40% A, 30% B); 17–20.5 min (50% B, 50% C); and 20.5–30 min (96% A, 2, B). 284 nm was used as the detecting wavelength (UV detector). The temperature of the column was maintained at 30 °C, and the injection size was 20 µL. By contrasting the retention times of compounds with those of real standards, compounds were identified. The chemical quantities were assessed using calibration curves and relative to list of the used standards (Supplementary Material 1).

### Antimicrobial activities

The agar diffusion method was employed to evaluate the antimicrobial activity of the beeswax sample against various microorganisms. This included Gram-negative bacteria such as *Klebsiella pneumoniae* (OR398655), *Escherichia coli* (OR398654), and *Enterobacter aerogenes* (OR398656), as well as Gram-positive bacteria like *Staphylococcus aureus* (OR398657), and unicellular fungi including *Candida albicans* (OR398658) and *Candida auris* (OR398659). The standard antimicrobial agents were of gentamicin (0.07 mg/mL) for bacteria and amphotericin B (0.4 µg/mL) for molds. As described by Kacaniova^14^, the antimicrobial activity was assessed by measuring the diameter of inhibition zones (mm).

### Determining the microorganisms’ minimal inhibitory concentrations (MICs)

To complete the MICs of *A. mellifera* beeswax, the procedure outlined in the recommendation was followed^15^. The MBC test was conducted on Mueller Hinton Agar (MHA) plates, and the MIC test was conducted using conventional broth microdilution techniques on a 96-well round-bottom microtiter plate. At 10^6^ CFU/mL, the microbial inoculums were concentrated. The microbiological inoculums, spanning from column 4 to column 12, were added to 100 μL of MHB together with 100 μL of crude ethanol extract of *A. mellifera* beeswax stock solution (800 mg/mL) for the MIC test.

We then diluted the inoculums twice. Column 4 had the maximum recorded material concentration on the microtiter plate, whereas Column 12 had the lowest concentration. Column 1, which contained the bacterial inoculums and the medium, served as a positive control. The medium was the only thing in Column 2, which acted as a negative control. After adding 30 μL of the resazurin solution to each well of the microtiter plate, the plate was incubated at 37 °C for a whole day. There were no discernible color shifts. To represent microbes, pink or colorless hues were utilized rather than blue or purple ones. The result was a blue color since the lowest possible concentration of the microorganism-growth-inhibiting agent (MIC) was used to get its value.

### Determination of minimum lethal concentrations (MLCs)

Ansari et al.^16^ employed a microbroth dilution experiment with slight changes to assess the MLCs of *A. mellifera* beeswax against the pathogens under investigation (All cultures were grown in media containing plant extract, the group in line number one without beeswax extract was taken as negative control and the group in line number two without bacteria was taken as positive control). A double dilution with different concentrations ranging from 200 to 1.56 mg/mL was chosen as the treatment to ascertain the MLCs. The overnight-grown cultures from every treatment concentration were streaked on agar plates to identify the MLCs.

### Methodology for making resazurin solution

According to Khalifa et al.^17^, a 0.02% (wt/vol) resazurin solution was produced. The 0.002 g of resazurin salt powder was dissolved in 10 mL of distilled water. The mixture was then vigorously mixed using a vortex. A Millipore membrane filter with a pore size of 0.2 μm was utilized to separate the mixture. The resazurin solution can be stored at 4 °C for 2 weeks.

### Anti-inflammatory assessment

#### Generating the suspension of erythrocytes

Blood was drawn from healthy individuals and placed in 3 mL heparinized tubes. The whole blood was then centrifuged for 10 min at approximately 3000 rpm. The red blood cell pellets were dissolved using an equivalent volume of saline, which was partially present in the supernatant. An isotonic buffer mixture (10 mM sodium phosphate buffer, pH 7.4) was used to create 40% v/v suspensions, after which the total volume of the disintegrated red blood cell pellets was calculated. The neutralizing solution contained 200 mg of NaH_2_PO_4_, 1115 mg of Na_2_HPO_4_, and 9 g of NaCl per liter of distilled water. The reconstituted supernatant, containing the patient’s newly regenerating blood cells, was then utilized.

#### Hemolysis triggered by hypotonicity

The hypotonic solution used in this investigation was prepared by dispersing beeswax in distilled water^18^. Beeswax concentrations (1000, 800, 600, 400, 200, and 100 μg/mL) were administered in duplicate pairs of centrifuge tubes, each containing 5 mL of an initially hypotonic solution. Additionally, to create a total of two sets of centrifuge tubes, beeswax doses (100–1000 μg/mL) were added to an aqueous isotonic solution (5 mL). The control set of tubes contained 5 ml of distilled water and 200 μg/mL of indomethacin. Each tube was thoroughly mixed with an additional 100 μL of erythrocyte suspension. After incubation for 1 h at room temperature, the solutions were centrifuged at 1300 rpm for 3 min. Hemoglobin levels were determined by measuring the absorbance (OD) of the supernatant at 530 nm using a Spectronic (Milton Roy) spectrophotometer. The percentage of hemolysis was calculated by comparing it to the hemolysis occurring in 100% distilled water. The following calculation showed that beeswax reduced hemolysis by:

$$\% {\text{Hemolysis inhibition}} = {1} - \left( {\left( {{\text{OD2}} - {\text{OD1}}} \right)/\left( {{\text{OD3}} - {\text{OD1}}} \right)} \right)*{1}00.$$

#### Cytotoxicity assay

A 96-well tissue culture plate was inoculated with 1 × 10^5^ Vero cells/mL (100 µL/well) using the MTT procedure, then incubated at 37 °C for 24 h to form a fully developed monolayer sheet, according to Riss and Moravec^19^. After the formation of a confluent cell monolayer, the 96-well microtiter plates’ growth medium was withdrawn, and the cell monolayer underwent two rounds of washing with wash media. To create a two-fold dilution series, a medium containing 2% serum (maintenance medium) was used. Three wells were left as controls, and they received just the maintenance medium. 0.1 mL of each dilution was added to each well. Following an incubation period of 37 °C, the plate was checked for physical indicators of toxicity, such as shrinkage, granulation, rounding of the cells, or partial or whole loss of the monolayer.

After adding 20 µL of MTT solution to each well, the plate was shaken vigorously for 5 min at 150 rpm to fully incorporate the MTT into the medium. After that, the plate was incubated for 1–5 h at 37 °C with 5% CO_2_ to allow the MTT to metabolize. Following incubation, any leftover medium was disposed of, and the plate was dried using paper towels. After being resuspended in 200 µL of DMSO, the formazan (MTT metabolic product) was vigorously mixed for 5 min at 150 rpm on a shaking table. In conclusion, optical density measurements were obtained at a wavelength of 560 nm, subtracting the background at 620 nm. This should result in a direct correlation with the number of cells.

#### Antiviral assay

To investigate the effect of beeswax on reducing the infectivity of Hepatitis A virus (HAV) and Coxsackie B4 virus (CoxB4), seed 10,000 cells in each well of a 96-well plate, using 200 µL of medium per well. Leave three wells as blank controls without any cells. Incubate the plate overnight at 37 °C with 5% CO_2_ to allow the cells to attach to the bottom of the wells. Then, mix a non-toxic concentration of the beeswax with an equal volume (1:1 v/v) of the viral solution. Add 100 µL of this mixture to each well and let it stand briefly. Agitate the plate at 160 rpm for 5 min on a shaker. Allow the virus to interact with the cells for 1 day at 37 °C with 5% CO_2_. Prepare approximately 2 mL of a 0.005 g/mL MTT solution in PBS for each microtiter plate. Add 20 µL of this MTT solution to each well. To ensure a thorough mixing of MTT with the culture medium, place the plate on a shaker and agitate at 160 rpm for 5 min. Incubate the plate with the MTT reagent at 37 °C and 5% CO_2_ for up to 5 h to facilitate metabolic activity. If necessary, remove the medium and gently blot the plate dry on tissue paper to eliminate any residues.

To dissolve the MTT metabolite Formazan, use 200 µL of DMSO per well. Agitate the plate on a shaker at 130 rpm for 5 min to effectively mix the formazan with the solvent. Measure the optical density at approximately 560 nm, correcting for any interference at 620 nm. A direct relationship between cell number and optical density should be observed^20^.

#### Antioxidant activity

Considering different concentrations of bio-generated beeswax (from 2000 to 31.25 μg/mL), the ability to scavenge DPPH radicals was evaluated. Using 95% ethanol, a solution of DPPH (2,2-diphenyl-1-picrylhydrazyl) radicals (1 mm) was prepared. After thoroughly shaking 200 μL containing beeswax concentration with 800 μL with DPPH solution, the mixture was left for 0.5 h, minutes at 25 °C in complete darkness. Centrifugation took place for 5 min at 13,000 rpm after that^21^. At 517 nm, the absorbance for every single concentration was evaluated in relation to a blank. The reference value was ascorbic acid. The equation has been employed to compute the DPPH scavenging activity (%) for standard and varied quantities of beeswax to assess their antioxidant activity:

$${\text{The scavenging capacity of DPPH }}\left( \% \right) = \left( {{\text{A1}} - {\text{A2}}} \right)/{\text{A1}}*{1}00$$

A1: Absorbance of control; A2: Absorbance of the sample.

### Statistical analysis

Chemical tests were carried out in triplicate, and the results were reported as mean ± standard deviations.

## Results and discussion

**Fig. 1:**
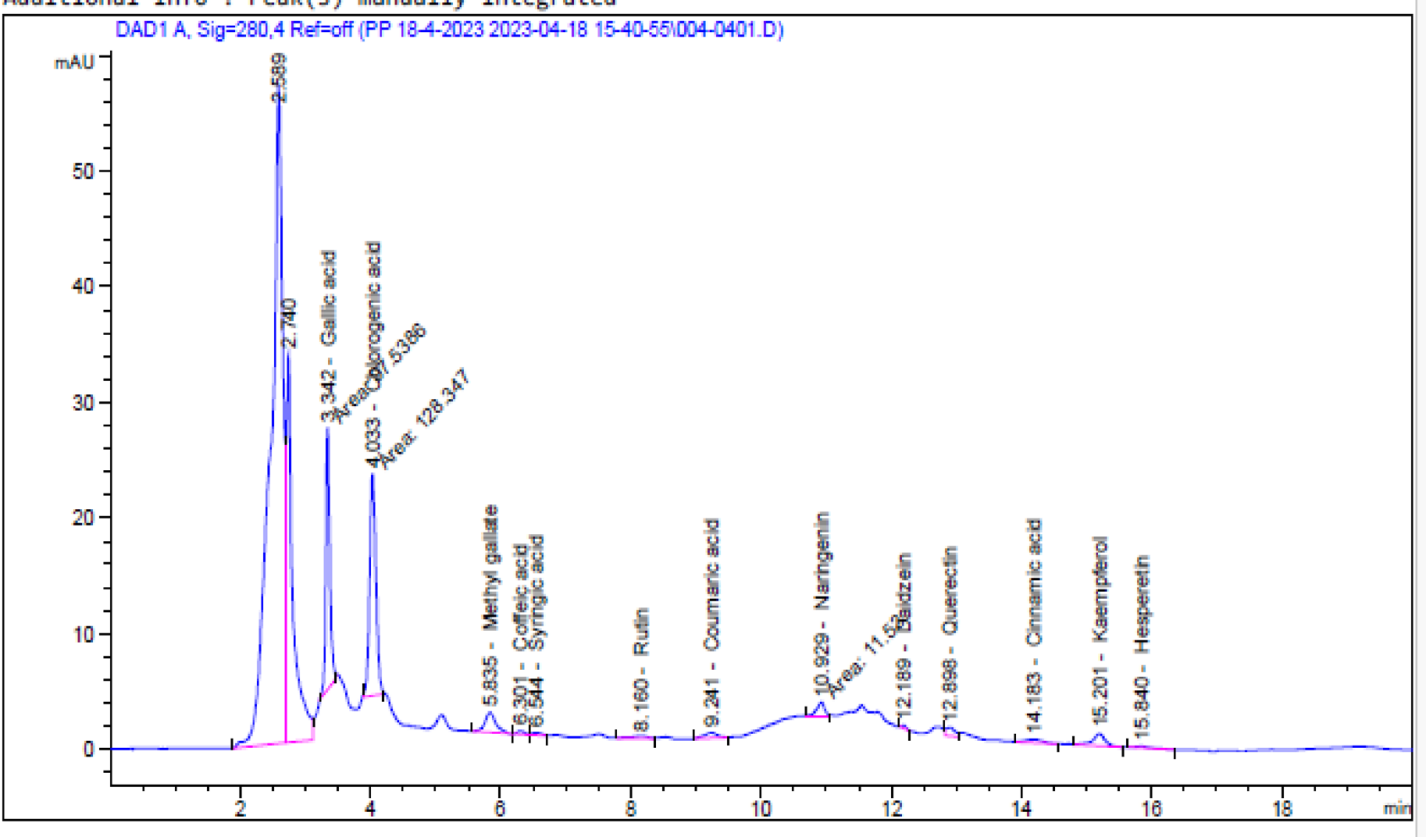
HPLC chromatogram shows the phenolic and flavonoid components found in the hydroalcoholic crude extract of *A. mellifera* found in beeswax.

**Fig. 2:**
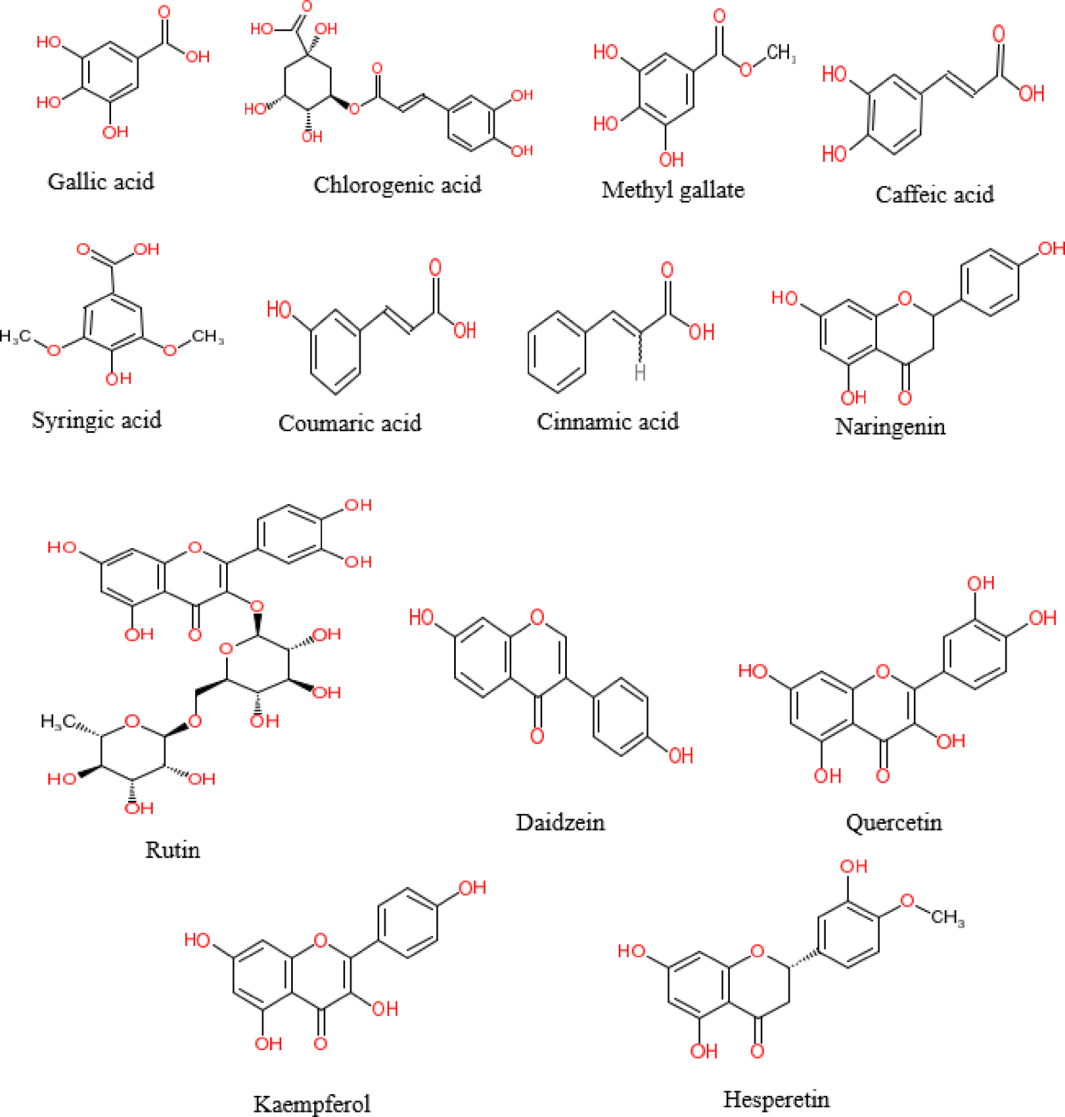
Phenolic structures identified of *A. mellifera* beeswax using HPLC.

A diverse combination of phenolic and flavonoid chemicals was revealed by HPLC analysis of the beeswax recovered from *A. mellifera*. The concentration and identification of thirteen phenolic compounds of *A. mellifera* wax extracted with 70% ethanol are shown in Table 1 and Fig. 1. This method detected the presence of several compounds in *A. mellifera* beeswax, including flavonoids such as rutin (19.39 µg/g), naringenin (69.52 µg/g), daidzein (3.34 µg/g), quercetin (53.69 µg/g), kaempferol (51.88 µg/g), hesperetin (8.13 µg/g), and phenolics such as gallic acid (421.13 µg/g), chlorogenic acid (878.80 µg/g), methyl gallate (56.21 µg/g), caffeic acid (8.83 µg/g), syringic acid (8.07 µg/g), coumaric acid (9.80 µg/g), and cinnamic acid (4.94 µg/g). The phenolic structures identified in *A. mellifera* beeswax using HPLC are illustrated in Fig. 2. Giampieri et al.’s research^22^, showed that leftovers from recycling beeswax are a rich source of proteins, minerals, and polyphenols, which greatly enhance overall antioxidant capacity while preserving low contaminant levels. This finding aligns with the conclusions drawn by Zhao et al.^23^.

**Table 1:** Analyzing the phenolic and flavonoid components in the ethanol extract of *A. mellifera* beeswax using high performance liquid chromatography.

Compounds	Concentration (µg/g)	Retention time (minutes)
Gallic acid	421.13	3.342
Chlorogenic acid	878.80	4.033
Methyl gallate	56.21	5.835
Caffeic acid	8.83	6.301
Syringic acid	8.07	6.544
Rutin	19.39	8.160
Coumaric acid	9.80	9.241
Naringenin	69.52	10.929
Daidzein	3.34	12.189
Quercetin	53.69	12.898
Cinnamic acid	4.94	14.183
Kaempferol	51.88	15.301
Hesperetin	8.13	15.840

Citrus beebread, one of the most valued honeybee products, was found to have a complex blend of flavonoid and phenolic components when it was extracted using high performance liquid chromatography (HPLC). The outcomes showed that sixteen phenolic compounds from citrus beebread that were extracted with 80% ethanol could be identified and their concentrations determined. While kaempferol is a minor flavonoid component with 34.02 µg/g, quercetin and rutin are the primary flavonoid compounds. The main phenolic components in citrus beebread extract are benzoic acid (852.20 µg/g), cinnamic acid (313.80 µg/g), P-hydroxy benzoic acid (253.6 µg/g), and ferulic acid (207.40 µg/g)^24^.

According to Sobral et al.^25^, quercetin, kaempferol, and myricetin were the primary phenolic components detected by HPLC. In the meantime, Isidorov et al.^26^, used GC–MS to identify phenolic compounds and found that the main flavonoids were quercetin, naringin, kaempferol, and caffeic acid, and the main phenolic compounds were 4-hydroxy benzoic, ferulic acid, and caffeic acid. According to Campos et al.^27^, pollen was responsible for the variance in phenolic compounds. The pollen’s origin and season can have an impact on the flora and its geographical location. According to several studies, the presence of phenolic chemicals like quercetin in beebread explains its anticancer and antioxidant properties^28,29^. Apart from their innate capacity to scavenge free radicals, many flavonoids also exhibit additional, particular functions. As an example, consider aromadendrin, a plant-based flavonoid with anti-inflammatory and anticancer properties that is significant in the pharmaceutical industry^30^. The solvent used in the extraction process determines whether active chemicals may be successfully extracted from the raw material. Given that flavonoids, aromatics, and polyphenolic chemicals make up most of the physiologically active components of propolis^31^, using solvents with a greater polarity can help increase yields and boost antioxidant activity.

Ghanem^32^ states that bacteria classified as Gram-positive (such as *S. aureus, S. epidermidis*, and *S. pyogenes*) and Gram-negative (like *P. aeruginosa, B. subtilis*, and *E. coli*) can be affected by beeswax exposure. Using a variety of bacterial strains (*S. aureus, S. epidermidis, B. subtilis, Salmonella enterica, E. coli,* and *L. monocytogenes*), as well as some yeasts (*C. albicans, C. tropicalis,* and *C. parapsilosis*) and molds (*A. niger, A. flavus*, and *A. fumigatus*), Kacániová et al.^33^ showed a high inhibitory activity of methanol and ethanol beeswax extracts. EL Sakka et al.^34^, Al-Waili^35^ and Abdulrhman et al.^36^, report that other authors looked at the antibacterial properties of beeswax when combined with other natural substances, including honey, propolis, and olive oil.

**Fig. 3:**
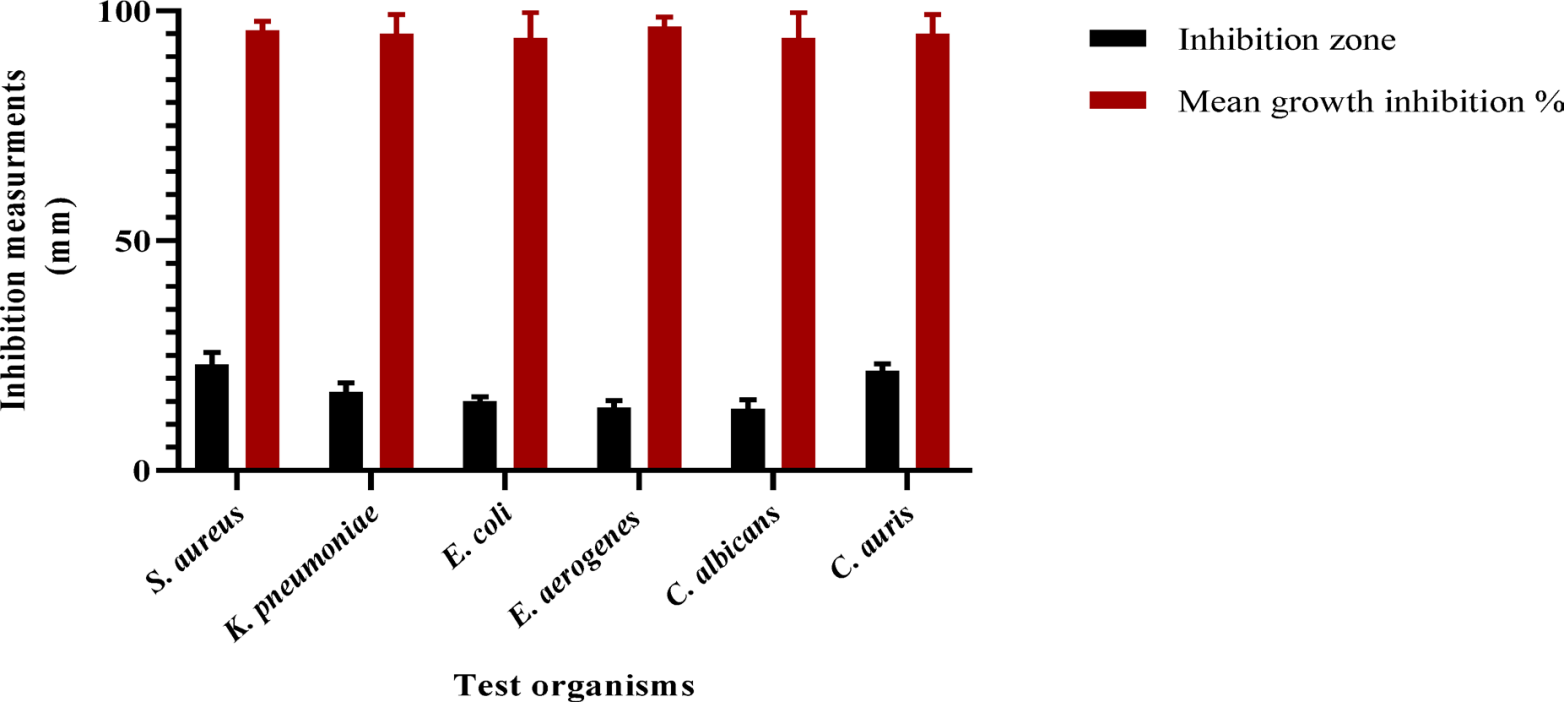
Antimicrobial activity histogram of *A. mellifera* beeswax (100 µg/mL) against the pathogenic strains using inhibition zone diameter (mm) and mean growth inhibition percentage (%) by agar well approach.

**Fig. 4:**
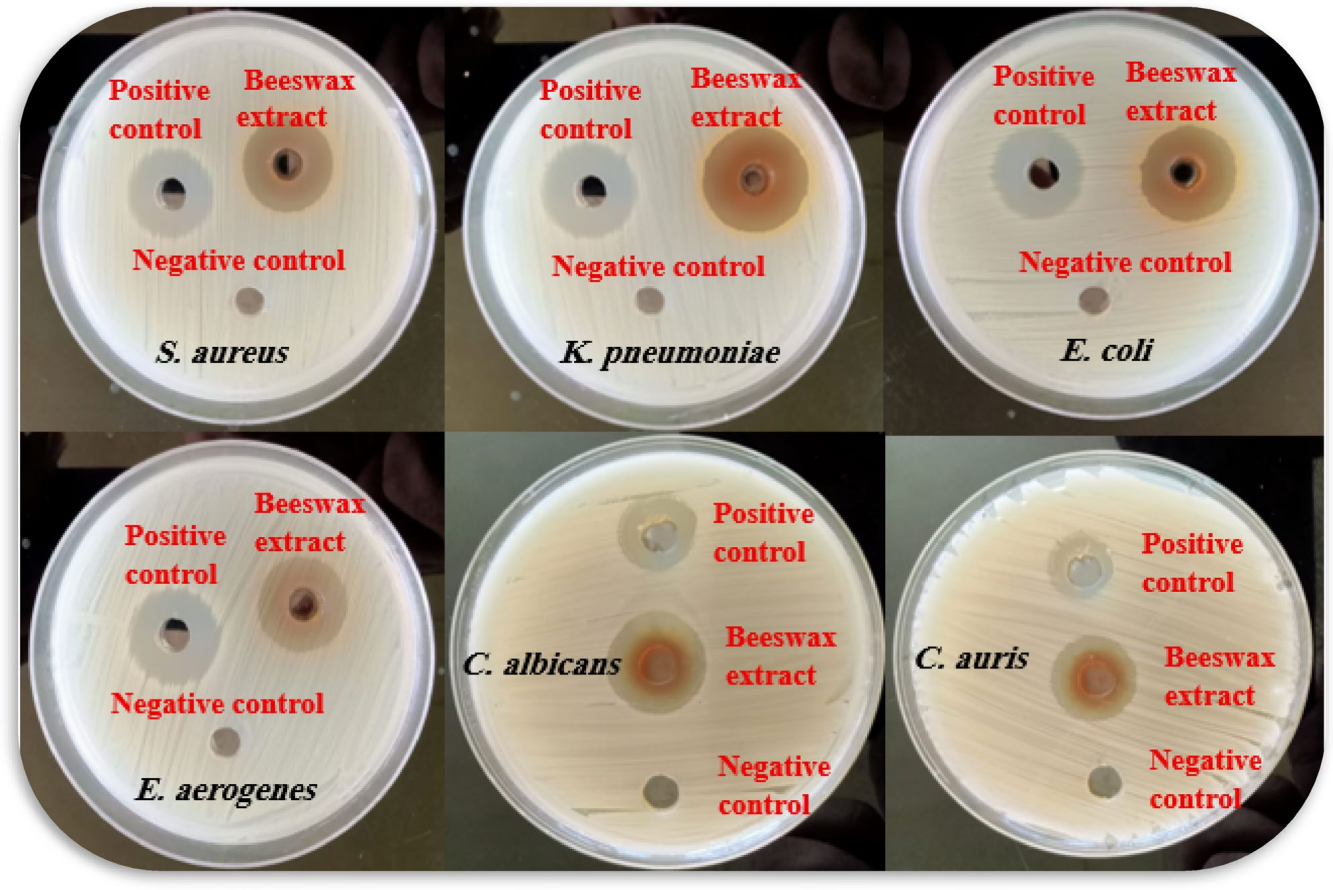
Antimicrobial activities of *A. mellifera* beeswax against *S. aureus*, *E. coli*, *C. albicans*, *C. auris*, *E. aerogenes*, and *K. pneumoniae.*

**Fig. 5:**
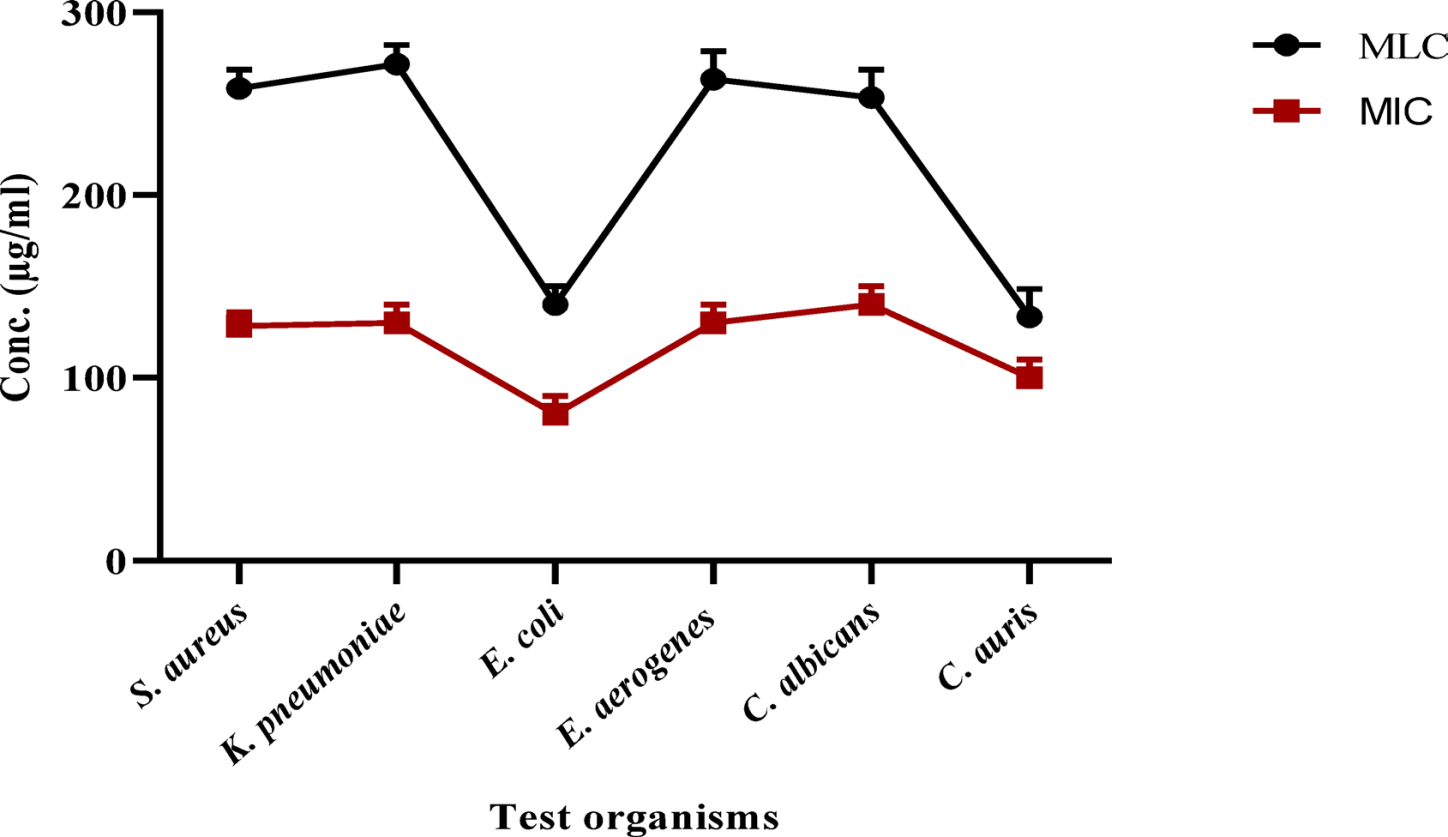
The *A. mellifera* beeswax’s minimal lethal concentrations (MLCs) and minimum inhibitory concentrations (MICs) against the pathogenic bacterial strains.

The antibacterial activity of a 70% ethanol beeswax extract was examined in the present investigation. The inhibition zone width (mm) of the beeswax extract against several pathogenic organisms is displayed in Table 2 and Figs. 3 and 4. According to the findings, beeswax had a greater inhibitory effect against *S. aureus* (25 ± 0.18 mm) and *E. coli* (15 ± 0.81 mm) than the reference chemical gentamicin. Furthermore, it showed significant inhibitory action against *C. auris* (22 ± 0.21 mm). Similarly, beeswax was shown to have moderate inhibitory action against *C. albicans* (15 ± 0.13 mm), *E. aerogenes* (14 ± 0.46 mm), and *K. pneumoniae* (19 ± 0.36 mm). Furthermore, Fig. 5 displays the *A. mellifera* beeswax’s minimal lethal concentrations (MLCs) and minimum inhibitory concentrations (MICs) against the pathogenic strains, which ranged from 125 to 250 and 62.5 to 125 μg/mL, respectively. After the incubation period, the color shift was examined. Colorlessness, or a change from purple to pink, indicates that the bacteria are actively metabolizing. The present results for antimicrobial activity of beeswax more likely related to chlorogenic acid as well as gallic acid as major compounds in accordance to report by to Sawicki et al.^37^, which reported the antimicrobial impact of phenolic molecules from bee products. The MICs and MLCs values for *S. aureus, K. pneumoniae, E. aerogenes*, and *C. albicans* were 125 μg/mL and 250 μg/mL, respectively. However, the MICs and MLCs for *E. coli* and *C. auris* were 62.5 μg/mL and 125 μg/mL, respectively.

**Table 2:** Antimicrobial activities of *A. mellifera* beeswax against different pathogenic microorganisms.

Pathogen	*A. mellifera* beeswax	Standard
Gram-positive bacteria		Gentamicin
*S. aureus* (OR398657)	25 ± 0.18	23 ± 0.81
Gram-negative bacteria		Gentamicin
*K. pneumoniae* (OR398655)	26 ± 0.36	24 ± 0.33
*E. coli* (OR398654)	22 ± 0.81	20 ± 0.75
*E. aerogenes* (OR398656)	19 ± 0.46	18 ± 0.25
Fungi		Amphotericin B
*C. albicans* (OR398658)	20 ± 0.13	16 ± 0.37
*C. auris* (OR398659)	22 ± 0.21	17 ± 0.75

Strong bacterial activity in beeswax suggested possible therapeutic effects, as previously described. Furthermore, compared to Gram-negative bacteria, Gram-positive bacteria exhibited a greater sensitivity to beeswax. This difference in sensitivity may be explained by the Gram-negative bacteria’s impermeable outer layer membrane^38,39^. This investigation supported the findings of Heesterbeek et al.^40^. Additionally, it appears that additional studies and testing have been conducted on the antibacterial properties of beeswax when combined with other natural goods and products from hives. Al-Waili^35^, reports that *S. aureus* and *C. albicans*, which are isolated from human patients, may be successfully suppressed from growing when honey, beeswax, and olive oil are combined 1:1:1:1, v/v. After the bacteria and yeast were incubated for 24 and 48 h at 37 °C, respectively, a zone of inhibition measuring 4 mm for *S. aureus* and 3.5 mm for *C. albicans* was seen^35^.

Propolis and beeswax have recently been studied for their antibacterial properties in a synergistic (1:1, v/v) ratio to prevent the development of *S. aureus* ATCC25923, *S. epidermidis* ATCC12228, *B. subtilis* ATCC27853, and *C. albicans* NCTC270^41^. Bees gather the resinous substance known as propolis from tree buds, and it is then processed with the help of enzymes, pollen, and beeswax. Because of its insulating qualities, bees utilize it as glue to seal their cell walls. Its usage in medicine goes back thousands of years because of its antibacterial, antiviral, antifungal, and anti-inflammatory effects^42^. The microorganisms that exhibited the highest sensitivity to the combination were *S. aureus* and *C. albicans*, with corresponding zones of inhibition of 20 and 22 mm^41^. Propolis and wax combined have been shown to exhibit antibacterial properties against *S. epidermidis* and *B. subtilis*, despite the latter two species’ minor resistance to the mixture (13.5 mm and 10.5 mm)^43^. The beebread extract’s antimicrobial properties are still present in flavonoid substances, including rutin, kaempferol, and quercetin. These substances block the production of adenosine triphosphate (ATP), halt ion channel exchange, and tear down the integrity of the bacterial cell wall^44^.

The immune system uses inflammation as a defensive mechanism in reaction to outside stimuli, including pathogens, toxic chemicals, bacterial infections, and radiation, to heal tissue damage and restore hemostasis. Various immune cells, including B and T lymphocytes, macrophages, monocytes, basophils and neutrophils, mast cells, and dendritic cells, are involved in the hallmark inflammatory processes of redness, swelling, heat, and pain^45^. Bees create a variety of goods, including venom, honey, propolis, royal jelly, pollen, and wax. Ancient Greeks, Chinese, and Egyptians have all attested to the nutritional, therapeutic, and health benefits of bee products^46^. Bee products have historically been utilized as nutritional supplements that promote health^47^.

**Fig. 6:**
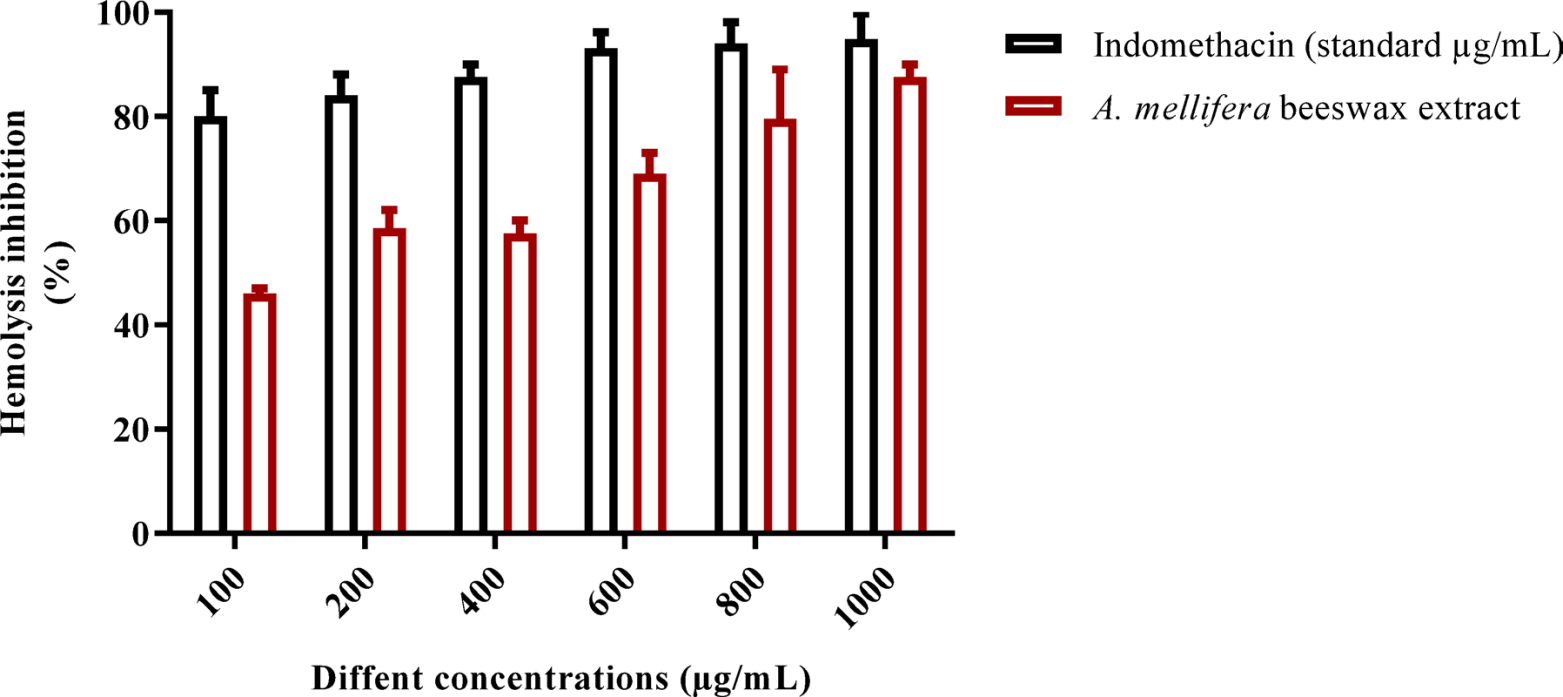
Effect of beeswax of hydroalcoholic crude extract of *A. mellifera* on HRBC hemolytic and membrane stabilization.

The anti-inflammatory effect of *A. mellifera* beeswax was evaluated by measuring its potential to generate hypotonicity-induced hemolysis and conducting a hemolytic assay in vitro, as shown by the results we obtained. The current investigation sought to evaluate the anti-inflammatory characteristics of *A. mellifera* wax at different concentrations between 1000 and 100 µg/mL, as seen in Fig. 6. The results showed that*,* as compared to indomethacin (standard drug), *Apis mellifera* beeswax had considerable anti-inflammatory activity percent hemolysis Inhibition was 90.2% at concentration 1000 µg/mL, while indomethacin (standard drug) showed 100% activity at the same concentration. These results support those of Nainu et al.^48^ and Ranneh et al.^49^, who discovered that bee products had a range of biological properties, such as anti-inflammatory, antibacterial, and antioxidant properties. Proteins, peptides, minerals, flavonoids, terpenes, fatty acids, and phenolic compounds are some of the physiologically active components found in bee products^1,47^.

**Fig. 7:**
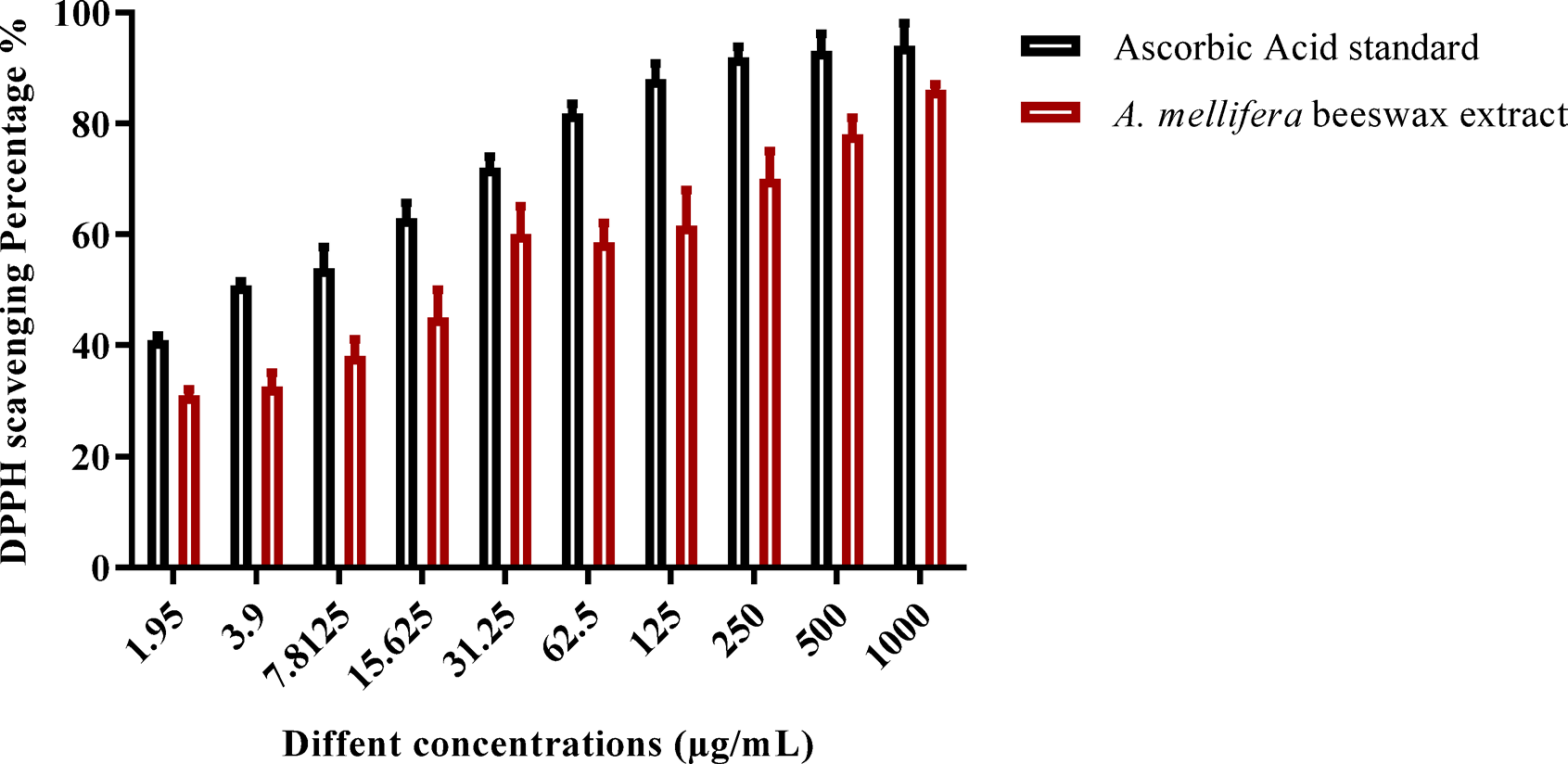
Antioxidant activity of hydroalcoholic crude extract of beeswax (*A. mellifera*).

The antioxidant activity showed considerable DPPH scavenging activity in the current study free radical scavenger activity tests. The antioxidant activity of *A. mellifera* beeswax using ascorbic acid as standard drug was evaluated at different concentrations from 1000 to1.95 µg/mL as shown in Fig. 7. Results showed that, as compared to ascorbic acid, *A. mellifera* beeswax had considerable antioxidant activity. Moreover, the IC_50_ of *A. mellifera* beeswax was 16.93 µg/mL.

Kamel et al.’s research^50^ indicates that the antioxidant activity (IC_50_) varied from the highest value (1748.25, 942.78 mg/mL in water extract and 1644.7, 532.280 mg/mL in ethanolic extract, respectively) found in Chinese and Egyptian propolis, pollen, and old wax comb samples to the lowest value found in sugar feeding honey and wax samples. Water extract had the greatest value, measuring 66.533, 36.625, 80.012, and 55.238 mg/mL; the other values were 13.878, 19.740, 51.625, and 36.108 mg/mL. Moreover, honeybee products’ ethanolic extract showed much stronger antioxidant activity than water extract. Nonetheless, there were no discernible differences in the antioxidant activity between the ethanolic and water extracts in the sugar fed honey or the wax sample.

Honey does not have as much antioxidant activity as bee bread, according to Baltrušaitytė et al.^51^. The antioxidant activity of bee bread was demonstrated using the IC_50_ values for DPPH (0.05 ± 0.01 mg/mL), ABTS (0.08 ± 0.05 mg/mL), and reducing power (0.05 ± 0.04 mg/mL). Sidor and colleagues looked at the antioxidant activity and total phenolic components in drone brood homogenate throughout different stages of the brood development process^52^. The larval phase exhibited the highest quantity of DPPH (20.5%), while the white-eyed pupae had the lowest level (6.3 to 70% ethanol extract). When *Trifolium alexandrinum* L. bee pollen ethanolic extract was compared to ethyl acetate, dichloromethane, and petroleum ether as solvents, the extract showed a higher ability to scavenge radicals. Dichloromethane had moderate DPPH scavenging capabilities (63%), whereas ethanolic extract demonstrated the highest activity (90%), followed by ethyl acetate (79%), and petroleum ether (75%)^39^.

According to Eraslan et al.^53^, the protein and phenolic chemicals as gallic acid and chlorogenic acid included in royal jelly exhibit strong antiradical activity (as measured by FRSA) against reactive oxygen species (ROS). The range of antioxidant activity found in this investigation might perhaps be attributed to the kind of solvent employed. The antioxidant activity of the ethanol (80%) extraction process was greater than that of the water extract. This might be the result of the concentration, polarity, and extraction solvents having an impact on the extracted extracts’ antioxidant activity. The components of bee products vary in structure; while hydrophilic components are more soluble in polar solvents like alcohols, hydrophobic components have a strong affinity for non-polar solvents like hydrocarbons. As a result, the composition of the extracts produced changes when different polar solvents are used. The concentration of bioactive chemicals in the extracts was shown to be affected differently by the various types of extraction solvents.

**Fig. 8:**
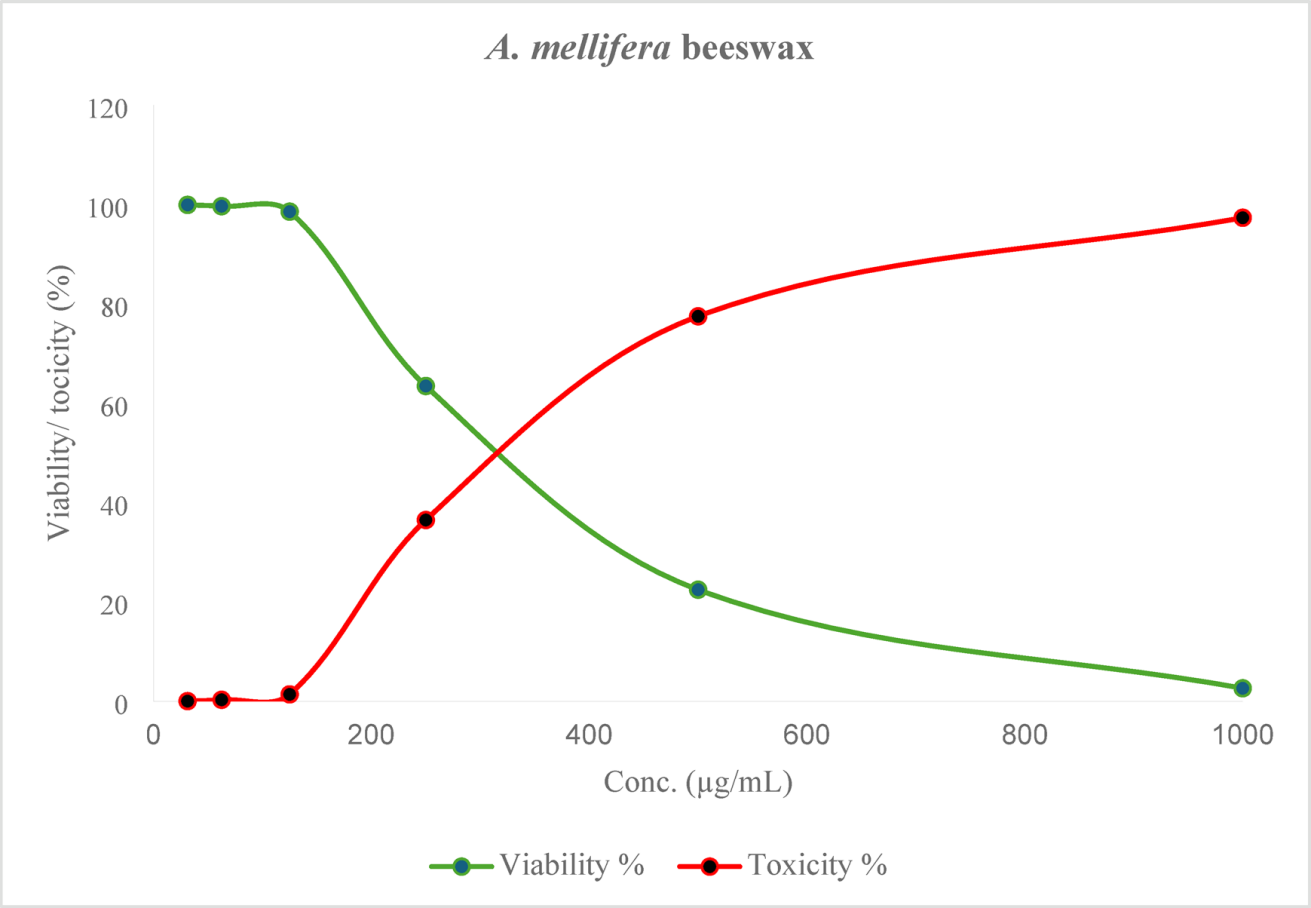
Effect of *A. mellifera* beeswax on Vero cells at different concentration.

**Fig. 9:**
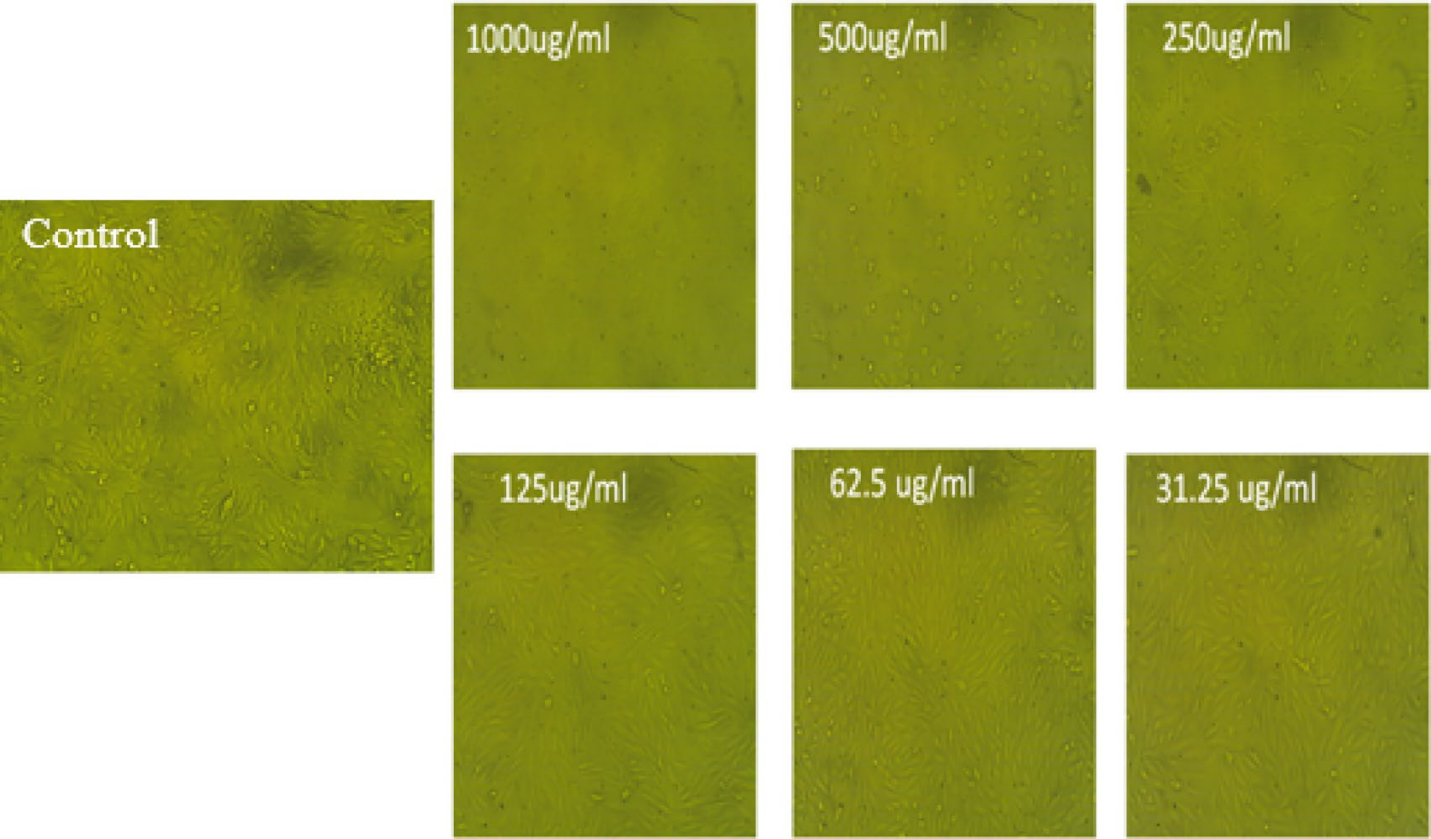
Morphological characteristic of Vero cells treated with *Apis mellifera* beeswax.

African green monkey kidney cells, treated as normal cells (Vero cells), were found to be toxically affected by *A. mellifera* beeswax. The MTT test was used to confirm the proportion of viable cells in the kidney at various doses between 1000 and 31.25 µg/mL at the conclusion of the incubation time. Figure 8 showed healthy cell death by significantly dose-dependent also when subjected to various quantities of* A. mellifera* beeswax. Based on the results, the half-maximal inhibitory concentration (IC_50_) for *A. mellifera* beeswax was found to be 348.84 ± 4.17 μg/mL. Figure 9 showed that change in cell shape after incubation of Vero cell with concentrations 1000–31.25 µg/mL imaged by inverted light microscope.

According to Jaganathan and Mandal^54^ and Carocho and Ferreira^55^, flavonoids have anticancer efficacy through distinct mechanisms of action, including the stimulation of tumor necrosis factors, apoptosis, and the inhibition of cell growth. Moreover, several investigations have documented that oleic acid and µ-linolenic acid can limit the growth of prostate cancer cells in various tumor cell lines^56^.

**Fig. 10:**
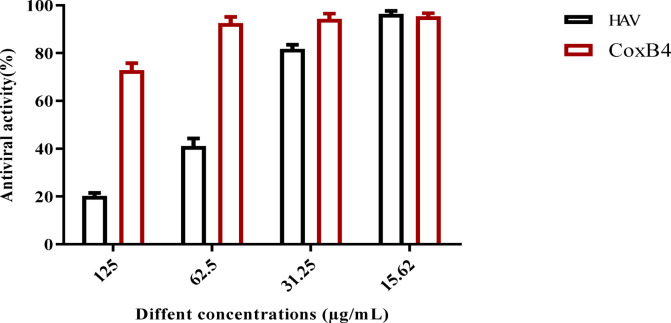
Antiviral assay of *A. mellifera* beeswax hydroalcoholic crude extract.

Studies have found that a deficiency in *A. mellifera* beeswax can significantly increase susceptibility to viral infections. One of the advantages of using *A. mellifera* beeswax for their antiviral properties includes their lower toxicity and enhanced effectiveness. As depicted in Fig. 10, the antiviral capabilities of *A. mellifera* beeswax extract were tested against human viruses, namely HAV and CoxB4. The maximum non-toxic concentration (MNTC) of *A. mellifera* beeswax, established at 125 µg/mL, was assessed using a standardized Vero cell line and was found to be significantly effective against these viruses. *A. mellifera* beeswax showed promising antiviral efficacy against both CoxB4 and HAV, with HAV being more susceptible. Specifically, the antiviral activity of *A. mellifera* beeswax against HAV was recorded at 78.52%, while it was 24.2% against CoxB4 at the same concentration. These findings demonstrate the potential of *A. mellifera* beeswax as effective antiviral agents against both HAV and CoxB4, underscoring their potential applications in biological contexts.

Bacterial and viral diseases are two of the world’s top causes of mortality. More and more reports detailing the emergence of bacterial and viral resistance, including in the form of polymicrobial infections, against currently available antibiotics and antivirals^57–59^ have recommended the use of substitute products with potential activity against those two types of pathogens.

Numerous studies^2,60,61^ indicate that one commodity connected to these activities is bee products. A wide range of traditional medicinal practices, encompassing the management of contagious diseases, have heavily relied on bee products such as honey, propolis, pollen, royal jelly, beebread, wax, and venom^62–64^.

Honey has been shown to have biological effects against bacterial pathogens and human pathogenic viruses, including the latest threat posed by SARS-CoV-2^65^. Most studies suggest that honey contains antiviral compounds with strong in vitro action against the varicella zoster virus (VZV) and the rubella virus^66,67^. Additionally, honey has been shown to have antiviral properties against the influenza virus, herpes simplex virus type 1 (HSV)-1, and respiratory syncytial virus (RSV)^68,69^. This is evident whether honey is consumed by itself or in conjunction with other goods. Moreover, honey has been demonstrated to increase lymphocyte proliferation and maintain optimal hematological and biochemical parameters, hence extending the life span of HIV-positive patients^35,68^.

A wide range of human pathogenic viruses, including human herpesviruses^70^, influenza viruses^71^, HIV^70^, human T-cell leukemia-lymphoma virus type 1 (HLTV-1)^72^, Newcastle disease virus (NDV)^73^, respiratory virus (RSV)^74^, poliovirus PV-type 1^75^, and dengue virus^76^, have also been demonstrated to be susceptible to propolis’ antiviral effects. Recently, quercetin, naringin, and as rutin three flavonoids present in propolis and honey have been suggested as potential adjuvant therapies for SARS-CoV-2^77^.

In addition to their antibacterial qualities, bee pollen and bread have been demonstrated to possess antiviral qualities. While date palm bee pollen was shown to be active against HSV-1 and HSV-2, bee pollen extracts from Korean papaver rhoeas were discovered to be relatively efficient against influenza viruses (strains of H1N1, H3N2, and H5N1)^78,79^. Bee pollen’s antiviral qualities were likely ascribed to flavonoids such as luteolin, galangin, kaempferol, and quercetin. Lutein is a potentially useful treatment option for influenza prevention since research has shown that it is one of the most potent neuraminidase inhibitors against the influenza virus^80^.

Moreover, it has been shown that quercetin inhibits the influenza virus’s ability to infect host cells by interacting with the HA2 component of hemagglutinin^78^. Quercetin-mediated hemagglutinin suppression may contribute to the prevention of the hemagglutinin-sialic acid interaction, which is required for influenza virus entry. Given the increasing prevalence of viral resistance to presently existing anti-influenza drugs, this mechanism will be critical to the pharmacological treatment of influenza virus infections in the future.

Researchers are becoming more interested in the pharmacological and medical uses of bee products with the identification of SARS-CoV-2, the agent that causes COVID-19, in late 2019. Numerous published studies have supported the use of bee products, including honey, propolis, pollen, bread, wax, and even venom, in the treatment of COVID-19. According to Lima et al.^81^, apitherapy is one of the alternative techniques that may be utilized to prevent and/or cure some of the symptoms associated with COVID-19. It has been shown that a number of the compounds in honey and other bee products are potent antivirals, which implies that they could be able to fight SARS-CoV-2^65,81^. This rationale has led to the present implementation of many randomized clinical trials to evaluate the effectiveness of propolis and honey in the treatment of COVID-19^81^. Propolis, honey, royal jelly, venom, and bee pollen are just a few of the honeybee products that have been the subject of an increasing amount of study on their antibacterial properties as possible natural medicines^41,82–84^. Only a few studies^32–34,36^ have confirmed beeswax’s antimicrobial qualities.

## Conclusion

Analyzing beeswax’s composition is crucial because, like other products made from beehives, the biological qualities of the substance, may be significantly impacted by the chemical variation of molecules from various botanic origins and/or geographic areas. In this work, a group of polyphenols, flavonoids could be detected in *A. mellifera* beeswax extract with potential antioxidant, antimicrobial and anti-inflammatory activity. Additionally, the extract has a strong inhibitory effect against CoxB4 and HAV. Thus, Egyptian bees wax extract could have applications in the food and pharmaceutical industries.

## Supplementary Information

Below is the link to the electronic supplementary material.

Supplementary Material 1

## Data Availability

The datasets used and/or analyzed during the current study available from the corresponding author on reasonable request.
