# Supplementary material for: Exploring the potential of hydro alcoholic crude extract of beeswax as antibacterial antifungal antiviral antiinflammatory and antioxidant agent
Source: Sci Rep. 2025 Sep 12;15:32512. doi: 10.1038/s41598-025-17830-4 (PMC12432216; doi:10.1038/s41598-025-17830-4)
Supplement: Supplementary file 1 — Supplementary Material 1 [file 41598_2025_17830_MOESM1_ESM.pdf]

Data File C:\CHEM32\1\DATA\PP 24-10-2023 2023-10-24 15-39-30\001-0101.D
Sample Name: Polyphenol STD

=====================================================================

Acq. Operator : SYSTEM  Seq. Line : 1
Acq. Instrument : hplc -2  Location : Vial 1
Injection Date : 10/24/2023 3:41:18 PM  Inj : 1
Inj Volume : 5.000 µl
Acq. Method : C:\CHEM32\1\DATA\PP 24-10-2023 2023-10-24 15-39-30\POLYPHENOL 2023.M
Last changed : 10/24/2023 3:39:30 PM by SYSTEM
Analysis Method : C:\CHEM32\1\DATA\PP 24-10-2023 2023-10-24 15-39-30\POLYPHENOL 2023.M ( Sequence Method)
Last changed : 10/25/2023 1:20:49 PM by SYSTEM
(modified after loading)
Additional Info : Peak(s) manually integrated

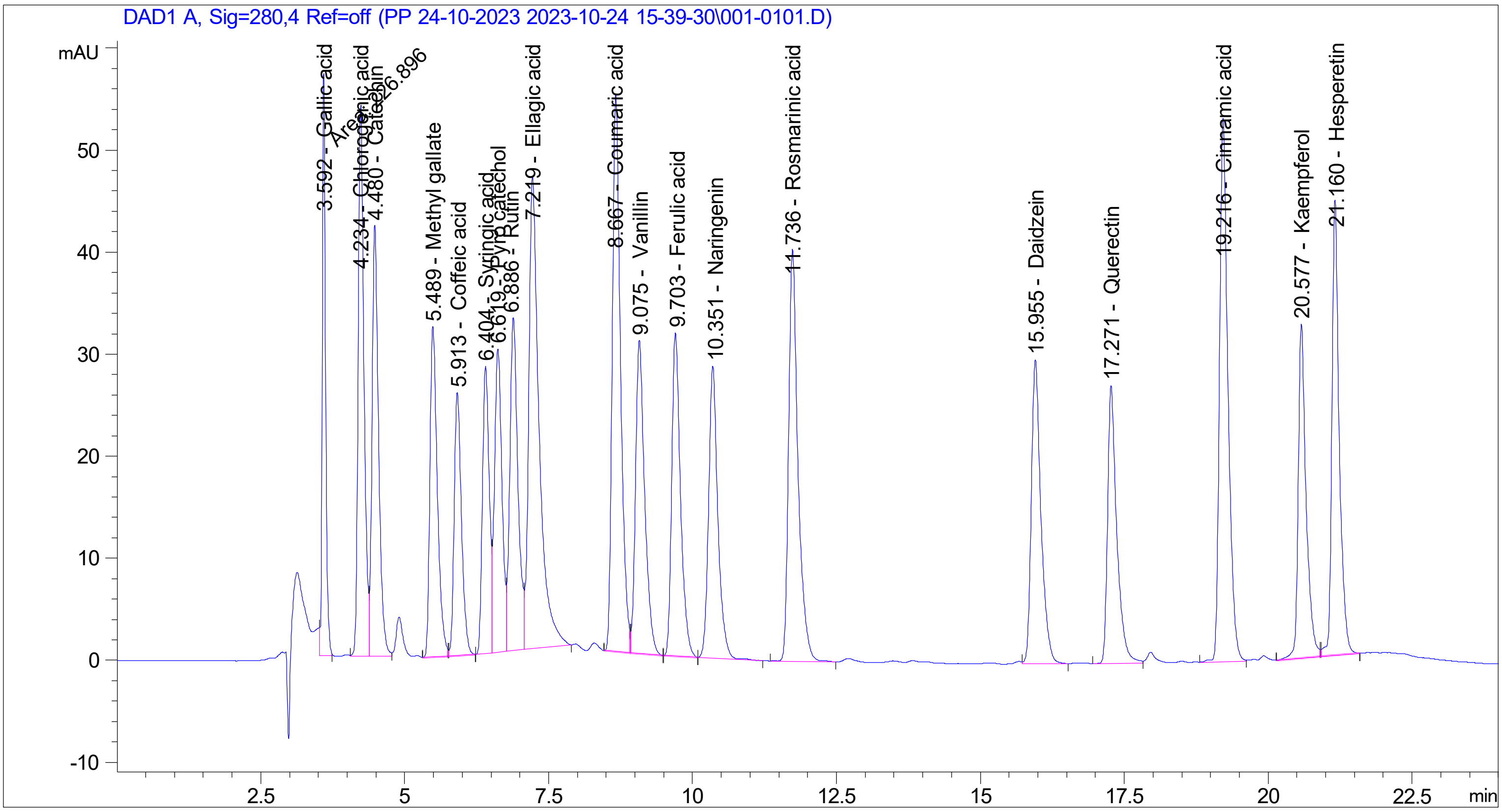


=====================================================================

Area Percent Report

=====================================================================

Sorted By : Signal
Calib. Data Modified : 10/25/2023 1:20:47 PM
Multiplier : 1.0000
Dilution : 1.0000
Use Multiplier & Dilution Factor with ISTDs

Signal 1: DAD1 A, Sig=280,4 Ref=off

| Peak # | RetTime [min] | Type | Width [min] | Area [mAU*s] | Area % | Name |
|---|---|---|---|---|---|---|
| 1 | 3.592 | MM | 0.0656 | 226.89642 | 3.3568 | Gallic acid |
| 2 | 4.234 | VV | 0.1054 | 377.19250 | 5.5803 | Chlorogenic acid |
| 3 | 4.480 | VV | 0.1187 | 335.67749 | 4.9661 | Catechin |
| 4 | 5.489 | BV | 0.1349 | 291.15628 | 4.3075 | Methyl gallate |
| 5 | 5.913 | VB | 0.1348 | 226.91400 | 3.3570 | Coffeic acid |

| Peak # | RetTime [min] | Type | Width [min] | Area [mAU*s] | Area % | Name |
|---|---|---|---|---|---|---|
| 6 | 6.404 | BV | 0.1255 | 230.51758 | 3.4104 | Syringic acid |
| 7 | 6.619 | VV | 0.1357 | 269.39828 | 3.9856 | Pyro catechol |
| 8 | 6.886 | VV | 0.1477 | 323.79147 | 4.7903 | Rutin |
| 9 | 7.219 | VB | 0.1832 | 583.23352 | 8.6286 | Ellagic acid |
| 10 | 8.667 | BV | 0.1509 | 547.69000 | 8.1027 | Coumaric acid |
| 11 | 9.075 | VB | 0.1648 | 339.60385 | 5.0242 | Vanillin |
| 12 | 9.703 | BB | 0.1593 | 335.24460 | 4.9597 | Ferulic acid |
| 13 | 10.351 | BB | 0.1684 | 320.28510 | 4.7384 | Naringenin |
| 14 | 11.736 | BV | 0.1685 | 458.62036 | 6.7850 | Rosmarinic acid |
| 15 | 15.955 | VB | 0.1713 | 345.52933 | 5.1119 | Daidzein |
| 16 | 17.271 | BV | 0.1610 | 300.84222 | 4.4508 | Querectin |
| 17 | 19.216 | BV | 0.1531 | 543.25385 | 8.0371 | Cinnamic acid |
| 18 | 20.577 | BV | 0.1385 | 310.08090 | 4.5875 | Kaempferol |
| 19 | 21.160 | VB | 0.1308 | 393.40155 | 5.8201 | Hesperetin |

Totals : 6759.32932

=====================================================================

*** End of Report ***

(Supplementary Figure 1) List of standards
